# Supplementary material for: Disruption of putrescine export in experimentally evolved Ralstonia pseudosolanacearum enhances symbiosis with Mimosa pudica
Source: mBio. 2025 Dec 2;17(1):e01225-25. doi: 10.1128/mbio.01225-25 (PMC12802222; doi:10.1128/mbio.01225-25)
Supplement: Text S1 — Supplemental methods. [file mbio.01225-25-s0001.docx]

**Supplemental materials and methods**

**Genome sequence analysis of evolved clones**

Single colonies from purified evolved clones were grown overnight in rich medium supplemented with trimethoprim. Bacterial DNA was extracted from 1 mL of culture using the Wizard genomic DNA purification kit (Promega). Evolved clone DNAs were sequenced at the GeT-PlaGe core facility (https://get. genotoul.fr/), INRAE Toulouse. DNA-seq libraries have been prepared according to Illumina’s protocols using the Illumina TruSeq Nano DNA HT Library Prep Kit. Briefly, DNA was fragmented by sonication, size selection was performed using SPB beads (kit beads), and adapters were ligated to be sequenced. Library quality was assessed using an Advanced Analytical Fragment Analyzer (Agilent), and libraries were quantified by quantitative PCR using the Kapa Library Quantification Kit (Roche). Sequencing has been performed on a NovaSeq6000 S4 lane (Illumina) using a paired-end read length of 2 × 150 bp with the Illumina NovaSeq Reagent Kits. Sequencing reads from NovaSeq6000 runs (all whole populations and clones from cycle 35) were mapped on the chimeric reference genome of the ancestral strain, comprising *R. pseudosolanacearum* GMI1000 chromosome (GenBank accession number: NC_003295.1) and megaplasmid (NC_003296.1) together with *C. taiwanensis* symbiotic plasmid pRalta (CU633751). Mutations were detected using breseq v0.33.1 (1) with default parameters using the consensus mode. Mutation lists were curated manually in order to remove mutations present in the ancestral strains as well as false-positive hits arising from reads misalignments in low complexity regions. Mutations detected in evolved clones represented in Fig. 2 are listed in Table S5.

**Phylogeny of evolved clones**

Based on the mutations that occurred during the evolution experiment, artificial sequences were constructed using only the positions mutated in the experiment and assigning each clone to the wild-type or the mutant allele based on its genome sequence. Phylogenetic trees were reconstructed online (www.phylogeny.fr)(2) using the maximum likelihood (ML) heuristic search under the LG model (3) with C-rate variation among sites (4), as implemented in PhyMLv3.0 (5).

**Mutant construction**

The *paeA*^V321G^ SNP mutants were constructed using the MuGent method described previously (6, 7). Briefly, a 6kb fragment carrying the point mutation was amplified by PCR using the G5 evolved clone as DNA matrix and the Phusion high-fidelity DNA polymerase (New England Biolabs). This fragment was co-transformed into competent strains of *R. pseudosolanacearum* strains together with a DNA fragment carrying an antibiotic resistance gene, either spectinomycin (pCBM142) or kanamycin resistance gene (pRCK-*PpsbA*-mCherry or pRCK-*PpsbA*-GFP), integrated into the neutral intergenic region downstream the *glmS* gene. Antibiotic-resistant transformants were selected and screened for the presence of the point mutation using PCR primer pairs specifically amplifying the wild-type or the mutant allele. The mutants were finally verified by Sanger sequencing of the mutated region.

Unmarked deletions of *paeA* and *nifH* were generated using the pEX18Tc plasmid carrying the *sacB* selection gene and the tetracycline resistance gene. Upstream and downstream fragments of the genes to be deleted were amplified by PCR using primers with overhanging *Bam*HI/*Xba*I and XbaI/HindIII restriction sites, digested with the appropriate restriction enzymes and cloned into the pEX18Tc *Bam*HI/*Hin*dIII digested plasmid. The resulting plasmid was introduced into strains of *R. pseudosolanacearum* by natural transformation and selection on tetracycline. The first integration event was verified by PCR. Transformants were then plated on rich medium supplemented with 5% saccharose to select for the second recombination event. Tetracycline sensitive and saccharose resistant clones were screened by PCR to identify the deleted mutants.

To complement the *paeA* mutants, the wild-type *paeA* gene was amplified by PCR using primers overhanging with *Bam*HI and *Hin*dIII restriction sites and a Phusion high-fidelity DNA polymerase (New England Biolabs). The PCR product was digested with the *Bam*HI and *Hin*dIII restriction enzymes and cloned into the pEX18Tc plasmid digested with the same enzymes. The resulting plasmid was integrated into the native *paeA* locus by natural transformation of *R. pseudosolanacearum* strains and subsequently excised by selection on rich medium supplemented with 5% saccharose. Complemented strains were identified by PCR screening and verified by Sanger sequencing.

To label *R. pseudosolanacearum* strains with a constitutively expressed *lacZ* gene, the P*psbA-lacZ* fusion from the pRCG-*PpsbA*-*lacZ* plasmid was integrated in the intergenic region downstream the *glmS* gene by natural transformation.

To introduce the p*nifH*-*gus* fusion, the *nifH* promoter region consisting of 668 bp upstream the start codon was amplified by PCR using primers overhanging with *Bam*HI and *Xba*I restrictions sites and a Phusion high fidelity DNA polymerase (New England Biolabs) and cloned upstream the promoterless *gus* gene of the pVO155 plasmid digested with the same enzymes. The resulting plasmid was introduced into *R. pseudosolanacearum* strains by triparental conjugation using the pRK600 as helper plasmid. Plasmid integration at the *nifH* promoter locus was verified by PCR.

Plasmids and PCR primers used for strain constructions are listed in Tables S4 and S6.

**Plant dry weights**

The aerial part of plants was harvested at 28 days post inoculation and dried at 65°C for two days. Three independent experiments were performed with 10 measurements per experiment and inoculated strain.

**Western blot visualization of NifH proteins**

*M. pudica* nodules were ground in liquid nitrogen, and then extracted in buffer (50 mM Tris-HCl, 150 mM NaCl, 10 mM EDTA, 0.2% triton, 2mM DTT, 1X protease inhibitor cocktail (Sigma)), sonicated 8 times 30 sec in ice and centrifuged at 4°C for 5 min at 16,000 g. Both supernatants and pellets were conserved. Since supernatants obtained from nodules formed by *Ralstonia* strains contained very low amounts of proteins, pellets were then subsequently lysed in Laemmli (2% SDS, 62.5 mM Tris HCl pH 6.8, 10% glycerol, 12.5 mM DTT, 0.005% Bromophenol blue). After centrifugation at room temperature at 16,000 g for 3 min, the supernatants were collected and proteins were quantified. Samples were heated at 95°C for 5 min and 15 µg were subjected to SDS-PAGE. A primary α-NifH antibody (Agrisera, AS01 021A) was used at a dilution of 1:1,500 and a secondary antibody Rabbit anti-Chicken IgY HRP conjugated (Agrisera, AS10 1489) was used at a dilution of 1:5,000 to examine the level of nitrogenase by Western blot. Equal loading was verified using a primary α-Tubulin antibody (Agrisera, AS10 680) at a dilution of 1:1,000 and a secondary antibody Goat anti-Rabbit IgG HRP conjugated (Agrisera, AS09 602) at a dilution of 1:10,000.

***M. pudica* RNA extraction and gene expression analysis**

Nodules were harvested at 10 days post-inoculation and ground in liquid nitrogen using a mortar and pestle prior to RNA extraction. To optimize the grinding, the powder was further mixed in a bead mill (Retsch MM400) for 30 sec 2 times at 30 Hz. Total plant RNA was isolated using the NucleoSpin RNA Plus kit (Macherey-Nagel) according to the manufacturer’s instructions, treated with a DNAse (Invitrogen) for 30 min at 37°C and then cleaned up with the NucleoSpin RNA clean-up kit (Macherey-Nagel). RNA quality was verified using a 2100 Bioanalyzer instrument (Agilent) and quantified using a QubitTM fluorometer (Thermo Fisher Scientific). cDNAs were synthesized from 1 µg of extracted RNA using a Transcriptor Reverse Transcriptase kit (Roche, Life technologies) and random hexamers as primers. Finally, *M. pudica* gene expression was measured using the Takyon® No ROX SYBR 2X MasterMix blue dTTP (Eurogentec) and the CFX Opus Dx Real-Time PCR Detection Systems (Bio-rad).

**Phylogenetic distribution of PaeA proteins**

The GenBank files corresponding to 1,255 bacterial genomes defined by Smith *et al.* (8) as representative of 3,498 RefSeq bacterial genomes were downloaded from the National Center for Biotechnology Information (NCBI) Genome Database, using scripts published by Smith *et al.* (8). *R. pseudosolanacearum* PaeA (RSc2277) protein homologs were extracted from the bacterial proteomes by BlastP (9). Proteins with at least 30% identity over 80% of the protein length were considered and mapped on the unrooted maximum-likelihood phylogenetic tree of 1,255 bacteria, inferred from the concatenated protein alignments of 31 single-copy proteins and published by Smith et al. (8). The complete list of genomes, in which PaeA homologs were detected, is provided in Table S2. The same scripts were modified to upload the reference genomes of all *Cupriavidus*, *Ralstonia* and *Pandorea* species available on NCBI, and the presence of PeaA homologs was analyzed with BlastP (Table S3).

**Supplemental references**

1. Deatherage DE, Barrick JE. 2014. Identification of mutations in laboratory-evolved microbes from next-generation sequencing data using breseq. *Methods Mol Biol* 1151:165-88.

2. Dereeper A, Guignon V, Blanc G, Audic S, Buffet S, Chevenet F, Dufayard JF, Guindon S, Lefort V, Lescot M, Claverie JM, Gascuel O. 2008. Phylogeny.fr: robust phylogenetic analysis for the non-specialist. *Nucleic Acids Res* 36:W465-9.

3. Le SQ, Gascuel O. 2008. An improved general amino acid replacement matrix. *Mol Biol Evol* 25:1307-20.

4. Yang Z. 1994. Maximum likelihood phylogenetic estimation from DNA sequences with variable rates over sites: approximate methods. *J Mol Evol* 39:306-14.

5. Guindon S, Dufayard JF, Lefort V, Anisimova M, Hordijk W, Gascuel O. 2010. New algorithms and methods to estimate maximum-likelihood phylogenies: assessing the performance of PhyML 3.0. *Syst Biol* 59:307-21.

6. Dalia AB, McDonough E, Camilli A. 2014. Multiplex genome editing by natural transformation. *Proc Natl Acad Sci U S A* 111:8937-8942.

7. Capela D, Marchetti M, Clérissi C, Perrier A, Guetta D, Gris C, Valls M, Jauneau A, Cruveiller S, Rocha EPC, Masson-Boivin C. 2017. Recruitment of a lineage-specific virulence regulatory Pathway promotes intracellular infection by a plant pathogen experimentally evolved into a legume symbiont. *Mol Biol Evol* 34:2503-2521.

8. Smith NT, Boukherissa A, Antaya K, Howe GW, Mergaert P, Rodríguez de la Vega RC, Shykoff JA, Alunni B, diCenzo GC. 2025. Taxonomic distribution of SbmA/BacA and BacA-like antimicrobial peptide transporters suggests independent recruitment and convergent evolution in host-microbe interactions. *Microb Genom* 11.

9. Camacho C, Coulouris G, Avagyan V, Ma N, Papadopoulos J, Bealer K, Madden TL. 2009. BLAST+: architecture and applications. *BMC Bioinformatics* 10:421.
