# Supplementary material for: Disruption of putrescine export in experimentally evolved Ralstonia pseudosolanacearum enhances symbiosis with Mimosa pudica
Source: mBio. 2025 Dec 2;17(1):e01225-25. doi: 10.1128/mbio.01225-25 (PMC12802222; doi:10.1128/mbio.01225-25)
Supplement: Supplemental Figures — Figures S1 to S6. [file mbio.01225-25-s0002.pdf]

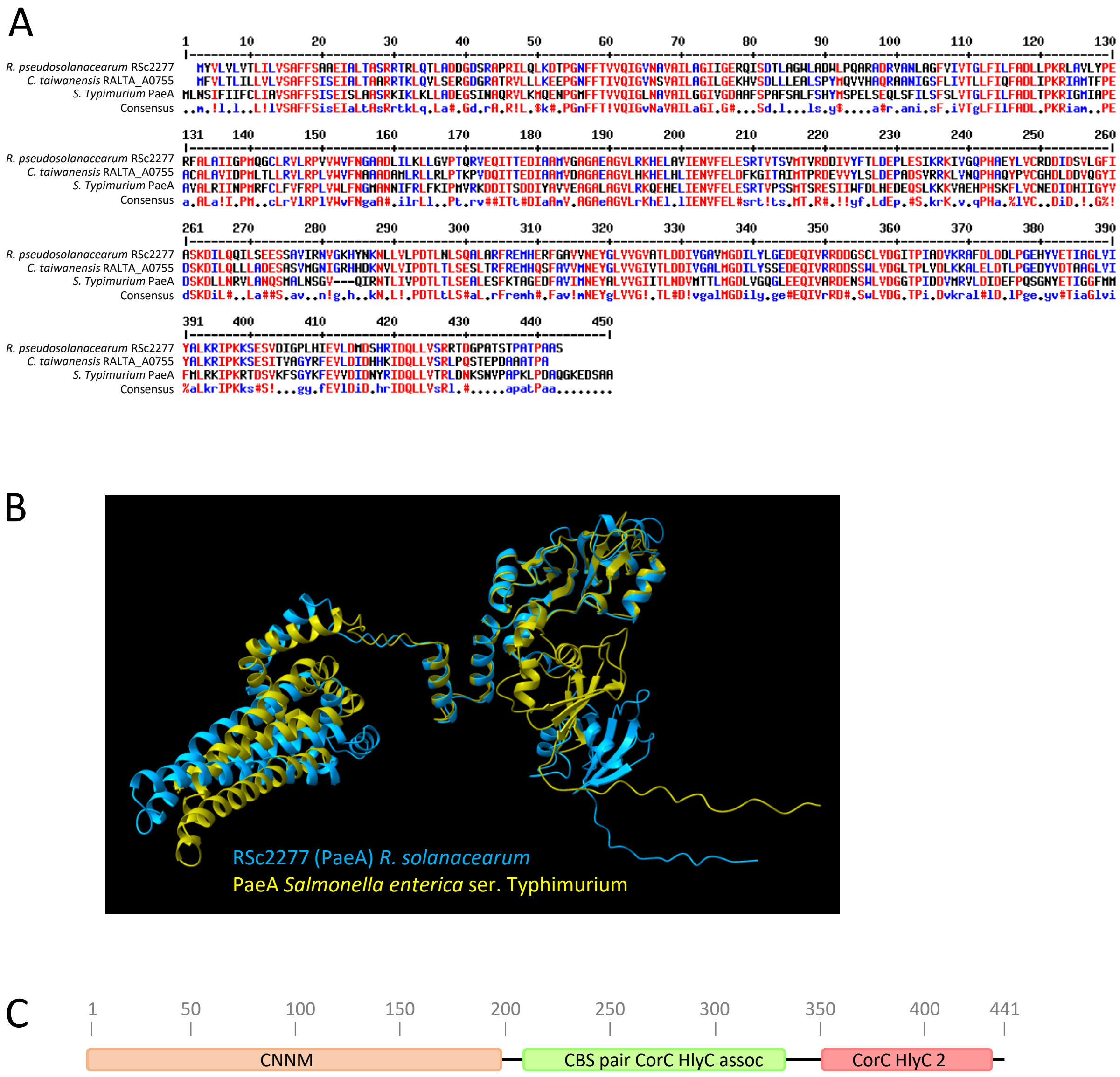

Fig. S1. A. Comparison of the PaeA protein sequences of *Ralstonia pseudosolanacearum* (RSc2277), *Cupriavidus taiwanensis* (RALTA_A0755) and *Salmonella enterica* ser. Typhimurium generated with Multalin (1). **B.** Three-dimensional structures of the PaeA proteins from *R. pseudosolanacearum* (in blue) and *S. enterica* ser. Typhimurium (in gold) predicted by AlphaFold (2) . Overlay generated by ChimeraX (3). The C-terminal region between amino acids 426 to 441 is a disordered region as predicted by the flDPnn software (4). **C**. InterPro scan domains of the *R. pseudosolanacearum* PaeA protein. CNNM, CBS-pair domain divalent metal cation transport mediator. CBS pair CorC HlyC assoc, two tandem repeats of the cystathionine beta-synthase (CBS pair) domains the majority of which are associated with the CorC_HlyC domain. CorC HlyC 2, domain that might be involved in modulating transport of ion substrates.

References

1. Corpet F. 1988. Multiple sequence alignment with hierarchical clustering. Nucleic Acids Res 16:10881-10890. https://doi.org/10.1093/nar/16.22.10881.
2. Jumper J, Evans R, Pritzel A, Green T, Figurnov M, Ronneberger O, Tunyasuvunakool K, Bates R, Žídek A, Potapenko A, Bridgland A, Meyer C, Kohl SAA, Ballard AJ, Cowie A, Romera-Paredes B, Nikolov S, Jain R, Adler J, Back T, Petersen S, Reiman D, Clancy E, Zielinski M, Steinegger M, Pacholska M, Berghammer T, Bodenstein S, Silver D, Vinyals O, Senior AW, Kavukcuoglu K, Kohli P, Hassabis D. 2021. Highly accurate protein structure prediction with AlphaFold. Nature 596:583-589. https://doi.org/10.1038/s41586-021-03819-2.
3. Meng EC, Goddard TD, Pettersen EF, Couch GS, Pearson ZJ, Morris JH, Ferrin TE. 2023. UCSF ChimeraX: Tools for structure building and analysis. Protein Sci 32:e4792. https://doi.org/10.1002/pro.4792.
4. Hu G, Katuwawala A, Wang K, Wu Z, Ghadermarzi S, Gao J, Kurgan L. 2021. flDPnn: Accurate intrinsic disorder prediction with putative propensities of disorder functions. Nat Commun 12:4438. https://doi.org/10.1038/s41467-021-24773-7.

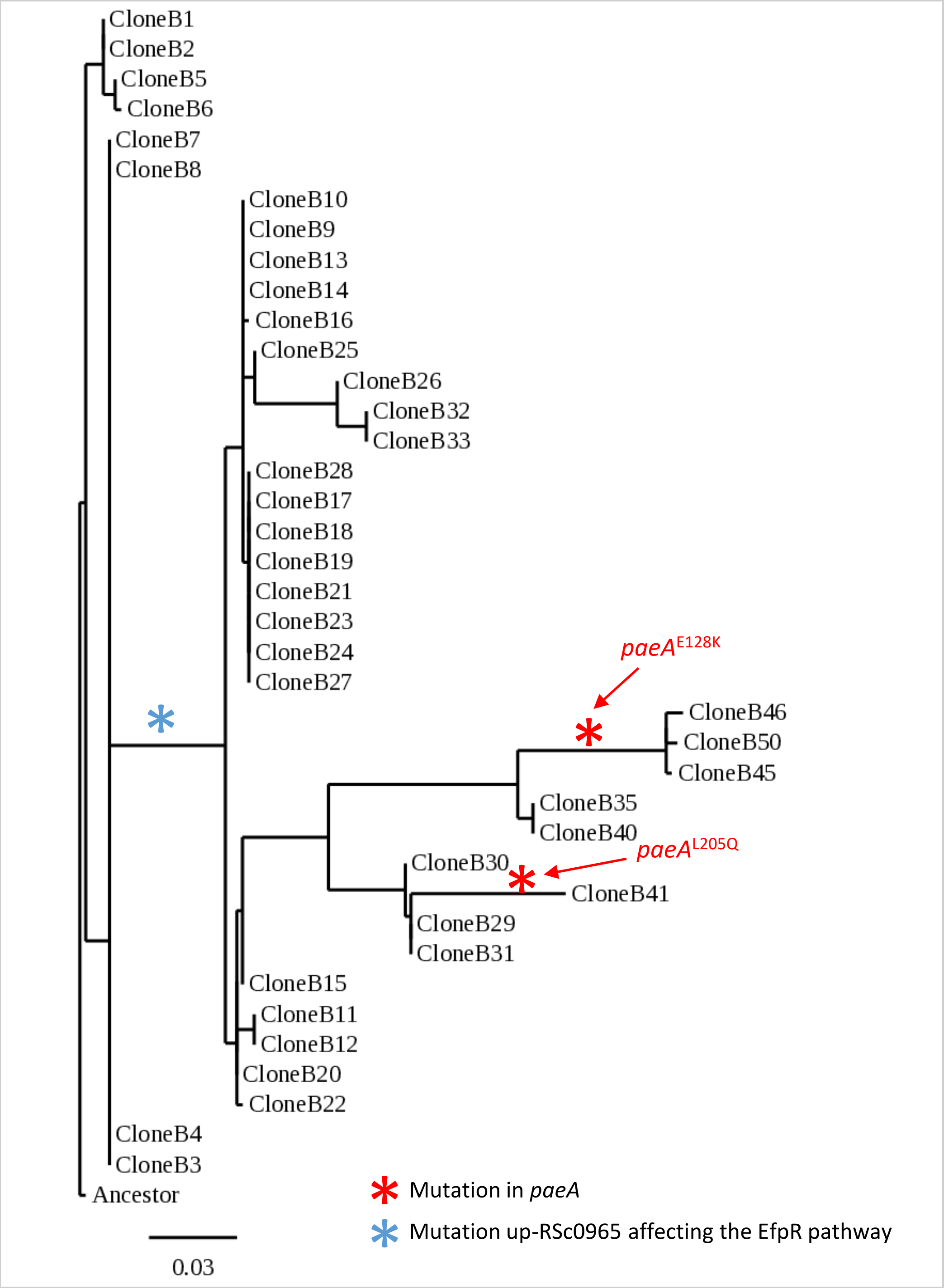

Fig. S2. Phylogenetic tree of evolved clones from the B lineage.
The tree was based on artificial sequences constructed by concatenating all the mutated allele positions occurring in the experiment. Maximum likelihood (ML) heuristic search under the LG model with C-rate variation among sites, as implemented in PhyMLv3.0 software, was used to construct the tree. The black bar represents the scale of genetic variation. Clone numbers indicate the cycle of the evolution experiment in which they were isolated. The blue star indicates the occurrence of the mutation up-RSc0965, which represses the EfpR regulator. The occurrence of mutations in the *paeA* gene is shown in red.

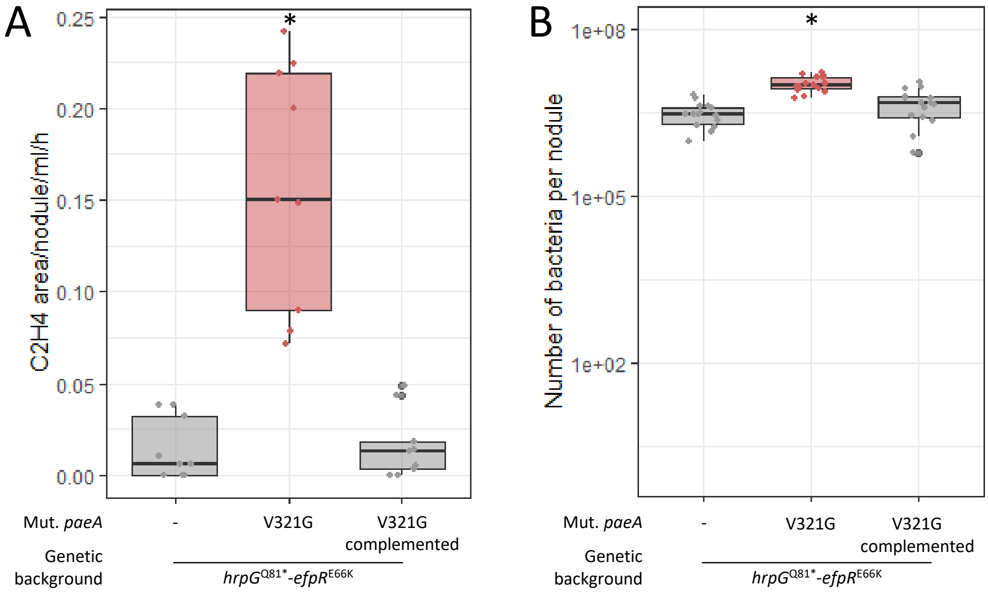

Fig. S3. Complementation of the *paeA*^{V321G} mutant in the *hrpG*^{Q81*}*-efpR*^{E66K} genetic background
Acetylene reduction assays (**A**) and number of viable bacteria recovered per nodule (**B**) measured on plants inoculated with the *hrpG*^{Q81*}*-efpR*^{E66K}*-paeA*^{V321G} mutant (red box plot) and its parental and complemented derived strains, 15 days post inoculation. * Statistically different from the parental strain ($P<0.05$, pairwise Wilcoxon test).

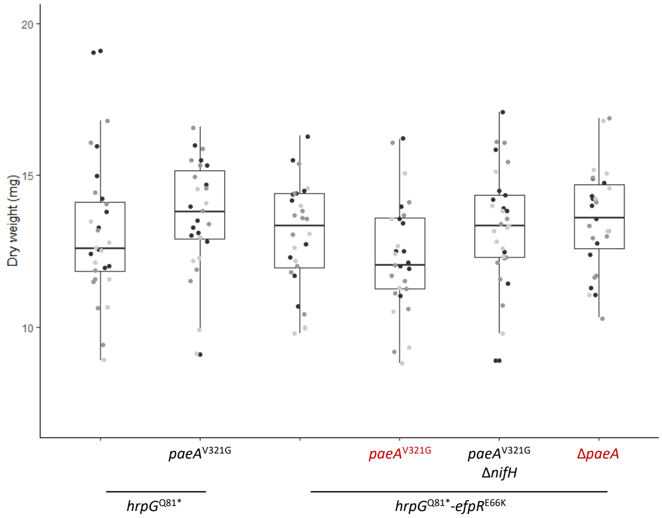

Fig. S4. Effect of *Ralstonia paeA* mutants on plant growth.
The aerial part of plants inoculated with *paeA* mutants in the *hrpG*^{Q81*} or *hrpG*^{Q81*}-*efpR*^{E66K} background was harvested at 28 dpi and dried at 65°C for two days. Three independent experiments were performed with 10 measurements per experiment and inoculated strain, displayed with three shades of grey. The dry weights of plants inoculated with strains showing detectable levels of nitrogenase activity (strains indicated in red) were not statistically different from the dry weights of plants inoculated with non-fixing strains (strains indicated in black) (*P*>0.05, ANOVA test).

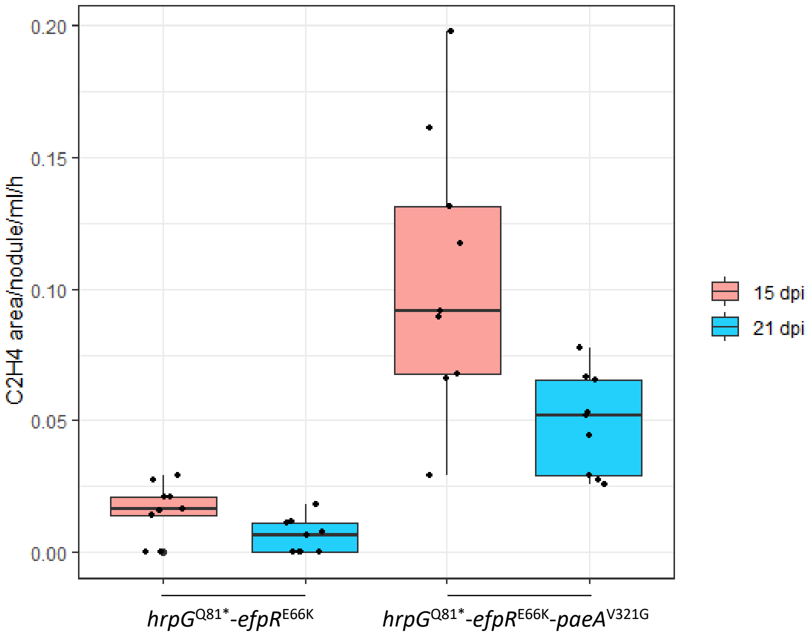

Fig. S5. Acetylene reduction assays at 15 and 21 days post-inoculation.
Plants were incubated with an excess of acetylene for four hours. Ethylene produced was measured by gas chromatography. Areas of ethylene peaks were integrated and normalized by the number of nodules, the volume of gas analyzed and the time of incubation with acetylene. Three independent experiments with three measures per experiment were performed for each strain.

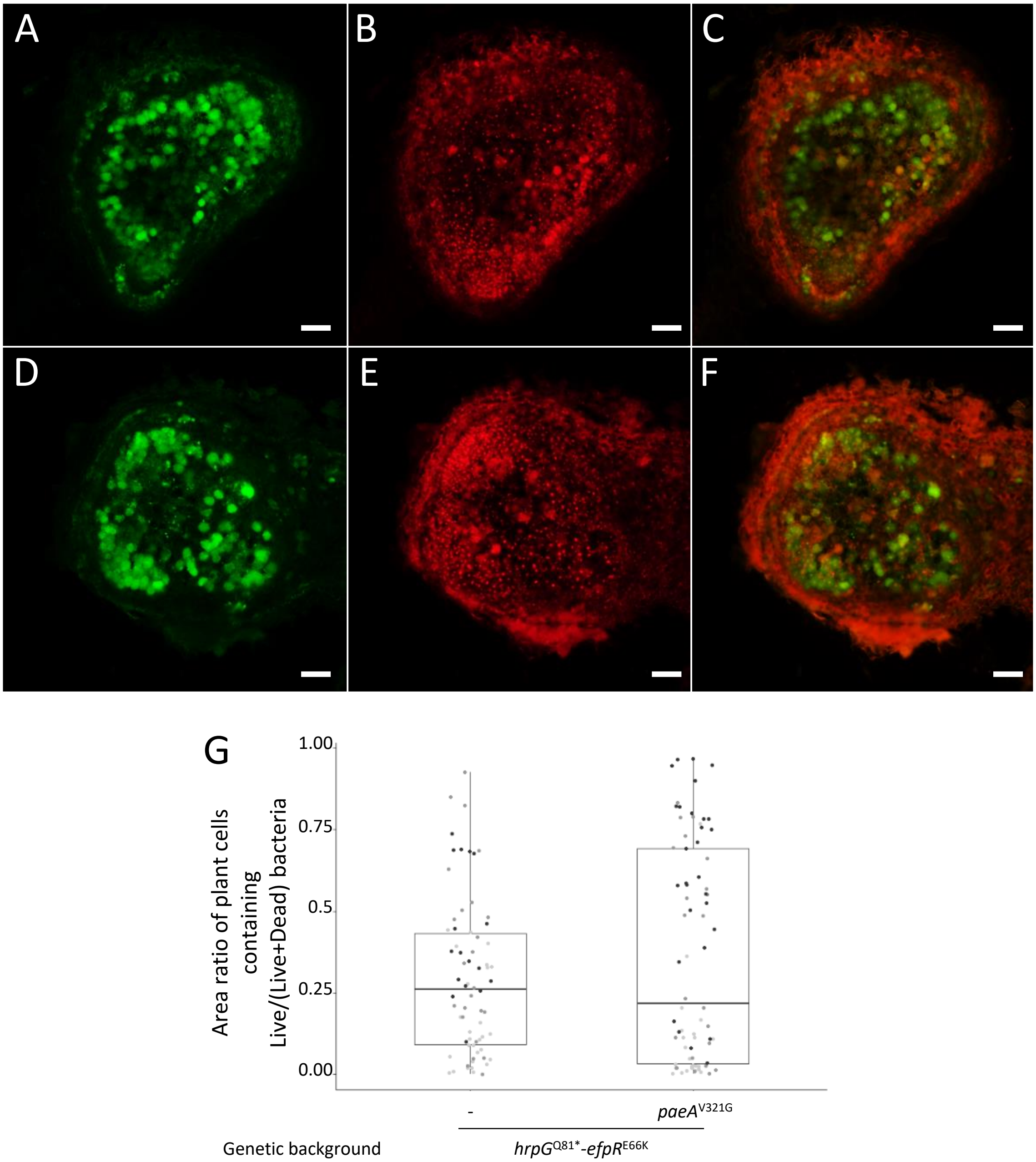

Fig. S6. **Intracellular persistence of bacteroids evaluated by LIVE/DEAD staining**

Sections of 15 day-old nodules formed by *R. pseudosolanacearum* GMI1000 pRalta *hrpG*^{Q81*} *efpR*^{E66K} (**A, B, C**) and its isogenic mutant *paeA*^{V321G} (**D, E, F**) were stained with the LIVE/DEAD™ BacLight™ Bacterial Viability Kit. (**A,D**) SYTO9 staining of live cells. (**B,E**) Propidium iodide staining of dead cells. (**C,F**) Overlay of SYTO9 and propidium iodide staining. White bars represent 100 µm length. (**G**) The area ratio of plant cells containing live bacteria to (live+dead) bacteria per nodule section was evaluated. At least 16 nodules per experiment and per strain were analysed in three independent experiments, shown as grey shades. Differences were not statistically significant (*P*=0.45, Wilcoxon test).
