# Supplementary material for: Disruption of putrescine export in experimentally evolved Ralstonia pseudosolanacearum enhances symbiosis with Mimosa pudica
Source: mBio. 2025 Dec 2;17(1):e01225-25. doi: 10.1128/mbio.01225-25 (PMC12802222; doi:10.1128/mbio.01225-25)
Supplement: Table S1 — Strain growth rates. [file mbio.01225-25-s0003.docx]

**Table S1.** **Growth rates of the *paeA* mutants and their parental strains in synthetic medium containing 10 mM glutamine as carbon source**

| Strain | µmax (h^-1^) | *P* value (*t*-test)* |
| --- | --- | --- |
| GMI1000 pRalta *hrpG*^Q81*^ | 0.266 ± 0.06 | 0.28459001 |
| GMI1000 pRalta *hrpG*^Q81*^ *paeA*^V321G^ | 0.226 ± 0.03 |  |
| GMI1000 pRalta *hrpG*^Q81*^ *efpR*^E66K^ | 0.236 ± 0.02 | 0.10538523 |
| GMI1000 pRalta *hrpG*^Q81*^ *efpR*^E66K^ *paeA*^V321G^ | 0.265 ± 0.02 |  |

*The *t*-test was performed between the growth rates of the *paeA* mutants and the corresponding parental strains.
