# Supplementary material for: Disruption of putrescine export in experimentally evolved Ralstonia pseudosolanacearum enhances symbiosis with Mimosa pudica
Source: mBio. 2025 Dec 2;17(1):e01225-25. doi: 10.1128/mbio.01225-25 (PMC12802222; doi:10.1128/mbio.01225-25)
Supplement: Table S4 — Strains and plasmids. [file mbio.01225-25-s0006.docx]

**Table S4.** Strains and plasmids used in this study.

| **Organism  Strain ID** | **Common name** | **Relevant characteristics** | **Reference/ source** |
| --- | --- | --- | --- |
| *E. coli* |  |  |  |
| DH5α | DH5α | *F recA lacZ*DM15 | Bethesda Research Laboratory |
| *R. solanacearum* |  |  |  |
| GMI1000 |  | wild-type strain (phylotype IA) isolated from tomato in French Guyana | (1) |
| RCM3804 | GMI1000 Δ*paeA* | Introduction of an unmarked deletion of *paeA* in GMI1000 | This study |
| RCM3438 | GMI1000 *paeA*^V321G^ IG*glmS*:P*psbA*-GFP | Introduction of the missense mutation *paeA*^V321G^ and a P*psbA*-GFP fusion downstream *glmS* in GMI1000, KanR | This study |
| *C. taiwanensis* |  |  |  |
| LMG19424 |  | Wild-type strain isolated from *Mimosa pudica* in Taiwan | (2) |
| CBM832 |  | LMG19424 derivative resistant to streptomycin, StrR | (3) |
| CBM2865 | CBM832 Δ*nifH* | Introduction of an unmarked deletion of *nifH* in CBM832, StrR | (4) |
| Chimeric *Ralstonia* | |  |  |
| CBM124 | GMI1000 pRalta | GMI1000 pRalta::Tri, TriR | (5) |
| CBM212 | Anc^B^ | CBM124-derived nodulating ancestor of the B lineage, mutated in *hrpG*^Q81*^, TriR, GenR | (5) |
| CBM349 | Anc^BK^ | CBM124-derived nodulating ancestor of the G and K lineages, mutated in *hrpG*^Q209*^, TriR, GenR | (5) |
| CBM356 | Anc^M^ | CBM124-derived nodulating ancestor of the M lineage, mutated in *hrcV*^Q589*^ (stop mutation), TriR, GenR | (5) |
| CBM1195 | B9 | Evolved clone from lineage B, cycle 9, TriR, GenR | (6) |
| RCM2305 | B30 | Evolved clone from lineage B, cycle 30, TriR, GenR | (7) |
| RCM2685 | B40 | Evolved clone from lineage B, cycle 40, TriR, GenR | This study |
| RCM2700 | B41 | Evolved clone from lineage B, cycle 41, TriR, GenR | This study |
| RCM3145 | B45 | Evolved clone from lineage B, cycle 45, TriR, GenR | This study |
| CBM465 | G2 | Evolved clone from lineage G, cycle 2, TriR, GenR | This study |
| CBM557 | G4 | Evolved clone from lineage G, cycle 4, TriR, GenR | (7) |
| CBM594 | G5 | Evolved clone from lineage G, cycle 5, TriR, GenR | (7) |
| CBM1125 | K4 | Evolved clone from lineage K, cycle 4, TriR, GenR | (7) |
| CBM1808 | K13 | Evolved clone from lineage K, cycle 13, TriR, GenR | (8), (7) |
| CBM597 | M5 | Evolved clone from lineage M, cycle 5, TriR, GenR | (6) |
| CBM643 | M6 | Evolved clone from lineage M, cycle 6, TriR, GenR | (7) |
| RCM3373 | X6 | Evolved clone from lineage X, cycle 6, TriR, KanR | This study |
| CBM1627 | GMI1000 pRalta *hrpG*^Q81*^ | Introduction of the stop mutation Q81* in *hrpG* in CBM124, TriR | (9) |
| RCM3467 | GMI1000 pRalta *hrpG*^Q81*^ *paeA*^V321G^ IG*glmS*:P*psbA*-GFP | Introduction of the missense mutation V321G in *paeA* and a P*psbA*-GFP fusion downstream *glmS* in CBM1627, TriR, KanR | This study |
| RCM3867 | RCM3467 *paeA* wild-type IG*glmS*:P*psbA*-GFP | Complementation of the RCM3467 strain with the wild-type allele of *paeA* introduced at the same locus | This study |
| RCM1865 | GMI1000 pRalta *hrpG*^Q81*^ *efpR*^E66K^ IG*glmS*:KanR*,* Anc^X^ | Introduction of the missense mutation E66K in *efpR* and a KanR cassette downstream *glmS* in CBM1627*,* TriR, KanR. Ancestor of the X lineage | (10) |
| RCM1866 | GMI1000 pRalta *hrpG*^Q81*^ *efpR*^E66K^ IG*glmS*:SpeR | Exhange of the kanamycin resistance cassette by a spectinomycin resistance cassette in RCM1865, TriR, SpeR | This study |
| RCM2940 | GMI1000 pRalta *hrpG*^Q81*^ *efpR*^E66K^ IG*glmS*:P*psbA*-*lacZ* | Exchange of the kanamycin resistance cassette by a P*psbA*-lacZ fusion downstream *glmS* in RCM1865, TriR, GenR | (11) |
| RCM3440 | GMI1000 pRalta *hrpG*^Q81*^ *efpR*^E66K^ *paeA*^V321G^ IG*glmS*:SpeR | Introduction of the missense mutation V321G in *paeA* and a spectinomycin resistance cassette downstream *glmS* in RCM1865, TriR, KanR | This study |
| RCM3475 | GMI1000 pRalta *hrpG*^Q81*^ *efpR*^E66K^ *paeA*^V321G^ IG*glmS*: P*psbA-*mCherry | Exchange of the spectinomycine resistance cassette by a P*psbA*-mCherry fusion downstream *glmS* in RCM3440, TriR, KanR | This study |
| RCM3642 | GMI1000 pRalta *hrpG*^Q81*^ *efpR*^E66K^ *paeA*^V321G^ IG*glmS*:P*psbA-lacZ* | Exchange of the P*psbA*-mCherry fusion by a P*psbA-lacZ* fusion downstream *glmS* in RCM3475, TriR, KanR | This study |
| RCM3871 | RCM3440 *paeA* wild-type | Complementation of the RCM3440 strain with the wild-type allele of *paeA* introduced at the same locus | This study |
| RCM3687 | GMI1000 pRalta *hrpG*^Q81*^ *efpR*^E66K^ *paeA*^V321G^ Δ*nifH* IG*glmS*:P*psbA*-mCherry | Introduction of an unmarked deletion of the *nifH* gene in RCM3475, TriR, KanR | This study |
| RCM3773 | GMI1000 pRalta *hrpG*^Q81*^ *efpR*^E66K^ Δ*paeA* IG*glmS*:KanR | Introduction of an unmarked deletion of *paeA* in RCM1865, TriR, KanR | This study |
| RCM3873 | GMI1000 pRalta *hrpG*^Q81*^ *efpR*^E66K^ pVO155:*nifH-GUS* IG*glmS*:SpeR | Genomic integration of the pVO155:*nifH*-GUS plasmid in the *nifH* promoter region in RCM1866 | This study |
| RCM3874 | GMI1000 pRalta *hrpG*^Q81*^ *efpR*^E66K^ *paeA*^V321G^ pVO155:*nifH-GUS* IG*glmS*:SpeR | Genomic integration of the pVO155:*nifH*-GUS plasmid in the *nifH* promoter region in RCM3440 | This study |
| RCM2495 | GMI1000 pRalta *hrpG*^Q81*^ *phcQ*^R154C^ IG*glmS*:P*psbA*-mCherry | Introduction of the missense R154C mutation in *phcQ* and a P*psbA*-mCherry fusion downstream *glmS* in CBM1627, TriR, KanR | (8) |
| RCM3820 | GMI1000 pRalta *hrpG*^Q81*^ *phcQ*^R154C^ Δ*paeA* IG*glmS*:P*psbA*-mCherry | Introduction of an unmarked deletion of *paeA* in RCM2495, TriR, KanR | This study |
| CBM125 | GMI1000pRalta *hrcV::Ω* | Inactivation of *hrcV* by a spectinomycin resistance cassette in CBM124, TriR, SpeR | (5) |
| RCM3854 | GMI1000pRalta *hrcV::Ω* Δ*paeA* | Introduction of an unmarked deletion of *paeA* in CBM125, TriR, SpeR | This study |
| CBM1619 | GMI1000pRalta *hrcS::Ω vsrA::Ω* | Inactivation of *hrcS* by a kanamycin resistance cassette and *vsrA* by a spectinomycin resistance cassette in CBM124, TriR, KanR, SpeR | (12) |
| RCM3857 | GMI1000pRalta *hrcS::Ω vsrA::Ω* Δ*paeA* | Introduction of an unmarked deletion of *paeA* in CBM1619, TriR, KanR, SpeR | This study |
| **Plasmid names** | **Relevant characteristics** | | **Reference** |
| pRalta | Symbiotic plasmid of LMG19424 (0.5 Mb) | | (13) |
| pEX18Tc | Suicide plasmid carrying the *sacB* gene, TetR | | (14) |
| pCBM142 | pGEM-T plasmid carrying the SpeR cassette inserted in the intergenic region downstream *glmS*, SpeR, AmpR | | (15) |
| pRCK-P*psbA*-GFP | Plasmid for *R. solanacearum* chromosomal integration of the constitutive *psbA* promoter fused to GFPuv into the intergenic region downstream *glmS*, KanR | | (10) |
| pRCK-P*psbA*-mCherry | Plasmid for *R. solanacearum* chromosomal integration of the constitutive *psbA* promoter fused to mCherry into the intergenic region downstream *glmS*, KanR | | (10) |
| pRCG-P*psbA*-lacZ | Plasmid for *R. solanacearum* chromosomal integration of the constitutive *psbA* promoter fused to *lacZ* into the intergenic region downstream *glmS*, GenR | | (16) |
| pVO155 | Integrative plasmid carrying the promoterless *gus* gene, KanR | | (17) |
| pVO155:*nifH*-*gus* | Integration of a 668 bp fragment corresponding to the *nifH* promoter region unstream the *gus* gene of pVO155 | | This study |
| pRK600 | Helper plasmid for triparental conjugation, ChlR | | (18) |

TriR, trimethoprim resistant. SpeR, spectinomycin resistant. GenR, gentamycin resistant. KanR, kanamycin resistant. TetR, tetracyclin resistant. AmpR, ampicillin resistance. ChlR, chloramphenicol resistance

**Supplemental references**

1. Boucher CA, Barberis PA, Trigalet AP, Demery DA. 1985. Transposon mutagenesis of *Pseudomonas solanacearum* – isolation of Tn5-induced avirulent mutants. *J Gen Microbiol* 131:2449-2457.

2. Chen WM, Laevens S, Lee TM, Coenye T, De Vos P, Mergeay M, Vandamme P. 2001. *Ralstonia taiwanensis* sp nov., isolated from root nodules of *Mimosa* species and sputum of a cystic fibrosis patient. *Int J Syst Evol Microbiol* 51:1729-1735.

3. Daubech B, Poinsot V, Klonowska A, Capela D, Chaintreuil C, Moulin L, Marchetti M, Masson-Boivin C. 2019. A new nodulation gene involved in the biosynthesis of Nod Factors with an open-chain oxidized terminal residue and in the symbiosis with. *Mol Plant Microbe Interact* 32:1635-1648.

4. Daubech B, Remigi P, Doin de Moura G, Marchetti M, Pouzet C, Auriac MC, Gokhale CS, Masson-Boivin C, Capela D. 2017. Spatio-temporal control of mutualism in legumes helps spread symbiotic nitrogen fixation. Elife 6:e28683. doi: 10.7554/eLife.28683.

5. Marchetti M, Capela D, Glew M, Cruveiller S, Chane-Woon-Ming B, Gris C, Timmers T, Poinsot V, Gilbert LB, Heeb P, Medigue C, Batut J, Masson-Boivin C. 2010. Experimental evolution of a plant pathogen into a legume symbiont. *PLoS Biol* 8.

6. Marchetti M, Jauneau A, Capela D, Remigi P, Gris C, Batut J, Masson-Boivin C. 2014. Shaping bacterial symbiosis with legumes by experimental evolution. *Mol Plant Microbe Interact* 27:956-64.

7. Doin de Moura GG, Mouffok S, Gaudu N, Cazalé AC, Milhes M, Bulach T, Valière S, Roche D, Ferdy JB, Masson-Boivin C, Capela D, Remigi P. 2023. A selective bottleneck during host entry drives the evolution of new legume symbionts. *Mol Biol Evol* 40.

8. Tang M, Bouchez O, Cruveiller S, Masson-Boivin C, Capela D. 2020. Modulation of quorum sensing as an adaptation to nodule cell infection during experimental evolution of legume symbionts. *mBio* 11.

9. Guan SH, Gris C, Cruveiller S, Pouzet C, Tasse L, Leru A, Maillard A, Médigue C, Batut J, Masson-Boivin C, Capela D. 2013. Experimental evolution of nodule intracellular infection in legume symbionts. *ISME J* 7:1367-77.

10. Capela D, Marchetti M, Clérissi C, Perrier A, Guetta D, Gris C, Valls M, Jauneau A, Cruveiller S, Rocha EPC, Masson-Boivin C. 2017. Recruitment of a lineage-specific virulence regulatory pathway promotes intracellular infection by a plant pathogen experimentally evolved into a legume symbiont. *Mol Biol Evol* 34:2503-2521.

11. Libourel C, Keller J, Brichet L, Cazalé AC, Carrère S, Vernié T, Couzigou JM, Callot C, Dufau I, Cauet S, Marande W, Bulach T, Suin A, Masson-Boivin C, Remigi P, Delaux PM, Capela D. 2023. Comparative phylotranscriptomics reveals ancestral and derived root nodule symbiosis programmes. *Nat Plants* 9:1067-1080.

12. Guan SH, Gris C, Cruveiller S, Pouzet C, Tasse L, Leru A, Maillard A, Medigue C, Batut J, Masson-Boivin C, Capela D. 2013. Experimental evolution of nodule intracellular infection in legume symbionts. *ISME J* 7:1367-1377.

13. Amadou C, Pascal G, Mangenot S, Glew M, Bontemps C, Capela D, Carrere S, Cruveiller S, Dossat C, Lajus A, Marchetti M, Poinsot V, Rouy Z, Servin B, Saad M, Schenowitz C, Barbe V, Batut J, Medigue C, Masson-Boivin C. 2008. Genome sequence of the beta-rhizobium *Cupriavidus taiwanensis* and comparative genomics of rhizobia. *Genome Res* 18:1472-1483.

14. Hoang TT, Karkhoff-Schweizer RR, Kutchma AJ, Schweizer HP. 1998. A broad-host-range Flp-FRT recombination system for site-specific excision of chromosomally-located DNA sequences: application for isolation of unmarked *Pseudomonas aeruginosa* mutants. *Gene* 212:77-86.

15. Remigi P, Capela D, Clerissi C, Tasse L, Torchet R, Bouchez O, Batut J, Cruveiller S, Rocha EP, Masson-Boivin C. 2014. Transient hypermutagenesis accelerates the evolution of legume endosymbionts following horizontal gene transfer. *PLoS Biol* 12:e1001942.

16. Monteiro F, Solé M, van Dijk I, Valls M. 2012. A chromosomal insertion toolbox for promoter probing, mutant complementation, and pathogenicity studies in *Ralstonia solanacearum*. *Mol Plant Microbe Interact* 25:557-68.

17. Oke V, Long SR. 1999. Bacteroid formation in the Rhizobium-legume symbiosis. *Curr Opin Microbiol* 2:641-646.

18. Finan TM, Kunkel B, Devos GF, Signer ER. 1986. Second symbiotic megaplasmid in *Rhizobium meliloti* carrying exopolysaccharide and thiamine biosynthesis genes. *J Bacteriol* 167:66-72.
